# Evaluation of different hydrocortisone treatment strategies in transsphenoidal pituitary surgery

**DOI:** 10.1007/s00701-019-03885-6

**Published:** 2019-05-07

**Authors:** Ola Fridman-Bengtsson, Charlotte Höybye, Laura Porthén, Pär Stjärne, Anna-Lena Hulting, Ola Sunnergren

**Affiliations:** 1 Department of Otorhinolaryngology, Karolinska University Hospital, Eugeniavägen 3, 171 76, Stockholm, Sweden; 2 Department of Clinical Sciences, Intervention and Technology, Division of Otorhinolaryngology, Karolinska Institute, Stockholm, Sweden; 3 Department of Molecular Medicine and Surgery, Patient Area Endocrinology and Nephrology, Inflammation and Infection Theme, Karolinska University Hospital, Stockholm, Sweden; 4 Department of Medicine, Västmanland County Hospital, Västerås, Sweden; 5 Department of Molecular Medicine and Surgery, Patient Area Endocrinology and Nephrology, Inflammation and Infection Theme, Karolinska University Hospital, Karolinska Institute, Stockholm, Sweden; 6 Department of Otorhinolaryngology, Ryhov County Hospital, Futurum– the Academy for Health and Care, Jönköping, Sweden

**Keywords:** Hydrocortisone, Transsphenoidal, Pituitary surgery, Serum cortisol, Short synacthen test, Hypocortisolism

## Abstract

**Background:**

Hydrocortisone treatment in transsphenoidal pituitary surgery has been debated. Although several publications advocate restrictive treatment, centers around the world administer stress doses of hydrocortisone in patients with presumed intact cortisol production. Our aim with this analysis was to compare postoperative hypocortisolism in patients who received three different protocols of hydrocortisone therapy during and after surgery.

**Method:**

This was a retrospective observational study. Based on perioperative hydrocortisone dose given, patients were divided in three groups: high dose (HD), intermediate dose (ID), and low dose (LD). Postoperative evaluation of the pituitary function was performed using S-cortisol at day 4 and short Synacthen test (SST) at 6–8 weeks. Patients with ACTH-producing adenomas or preoperative hydrocortisone treatment were excluded.

**Result:**

There was no difference between the groups regarding failure rate of SST. The rate of failed SST (all groups) was 51/186 (27%), 24/74 (32%) in the HD group and 26/74 (35%) and 11/38 (29%) in the ID and LD groups respectively. There was no significant difference between the ID and LD groups regarding S-cortisol at postoperative day 4 regarding serum cortisol level below 200 nmol/L. There was a significant but weak correlation, *r*_s_ 0.330 (*P* < 0.01) between S-cortisol day 4 and SST at 4–6 weeks.

**Conclusions:**

Peri and postoperative hydrocortisone treatment did not affect SST response 6–8 weeks postoperatively, whereas the rate of patients with S-cortisol below 200 nmol/L at postoperative day 4 did. LD hydrocortisone therapy seems to favor a better endogenous production in the early postoperative phase.

## Introduction

The necessity of perioperative corticosteroid treatment during pituitary surgery has been debated over the years [7, 10, 15, 19, 21, 25, 26, 27]. In the 1950s, Fraser et al. and Lewis et al. among others presented case reports with fatal outcome in patients with impaired hypothalamus-pituitary-adrenal (HPA) axis undergoing surgery without cortisone treatment [6, 13]. As a result of these reports, a strategy of giving stress doses of cortisone to all patients undergoing pituitary surgery was introduced [4, 8, 15, 20, 23].

In 2002, Inder et al. published treatment guidelines on cortisone therapy during and after pituitary surgery [7]. These guidelines were based on retrospective studies and suggested restricted cortisone treatment to patients with intact or presumed intact function of the HPA axis. Since 2002, few prospective studies have been published supporting the safety of a more restrictive use of cortisone treatment in pituitary surgery [11, 26]. Furthermore, one study even indicated that patients with impaired hypothalamic-pituitary-adrenal (HPA) axis managed without exogenous supply of cortisone during pituitary surgery [5].

Despite the 2002 guidelines, high doses of cortisone are still used in patients undergoing pituitary surgery in some centers around the world. One of the reasons may be difficulties in diagnosing and defining a suboptimal function of the HPA axis [1, 3, 24]. Other reasons may be local traditions—but also fear of inadequate pituitary function triggered by pituitary surgery.

At the Karolinska University Hospital, treatment schedules with high doses of hydrocortisone (i.e., a total of 970 mg during the week of surgery) were until 2010 used in all patients undergoing pituitary surgery, irrespective of the function of the HPA axis. In 2010, the postoperative hydrocortisone treatment was reduced due to a high frequency of reported symptoms of hypercortisolism. Then, after evaluation of the reduced hydrocortisone doses in November 2016 a further reduction of the perioperative cortisol dose was introduced in line with current international trends.

The aim of this study was to describe and evaluate postoperative morning S-cortisol and cortisol reservoir after transsphenoidal endoscopic pituitary surgery in relation to different hydrocortisone treatment schedules.

## Methods and materials

## Study design

This was a retrospective observational study based on data from medical records of patients with non-ACTH producing adenomas nor preoperative hydrocortisone treatment, who underwent transsphenoidal endoscopic pituitary surgery at the Karolinska University Hospital. Based on the perioperative hydrocortisone dose used, patients were divided in three groups: high dose (HD), intermediate dose (ID), and low dose (LD) (Table 1). Patients in the HD group were included 2008 to 2009, patients in the ID group 2010 to 2012, and patients in the LD group 2016 to 2017.

**Table 1:** Hydrocortisone treatment in the different groups

	HD	ID	LD
Day of surgery	300 mg*	300 mg*	150 mg*
POD 1	200 mg*	120 mg**	60 mg**
POD 2	120 mg**	50 mg**	30 mg**
POD 3	80 mg**	***	***
POD 4	80 mg**		
POD 5	60 mg**		
POD 6	30 mg**		

*POD 1*, first day after the day of surgery

*Intravenous hydrocortisone (Solucortef©)

**Oral hydrocortisone

***Dosage according to the morning S-cortisol level.

Analyses of blood samples were performed at the Department of Clinical Chemistry, Karolinska University Hospital, using electrochemical luminescence, (Modular E, Roche Diagnostics, Mannheim, Germany) who also supplied normative data. Due to a shift of reagents in 2016, S-cortisol values from HD and ID groups were recalculated in accordance with given instructions for comparison. As shown in Table 1, all patients operated during the period when the HD cortisol treatment schedule was used were prescribed cortisol for several weeks after surgery, i.e., until the short synacthen test (SST) at follow-up. S-cortisol was not analyzed in the HD group during the postoperative period. During the periods where ID and LD cortisol schedules were used, a morning S-cortisol was measured postoperatively at day 4 and used as an indicator for the need of continuous hydrocortisone treatment. An arbitrary level of < 200 nmol/L was routinely used as cutoff in assessing the risk of hypocortisolism. Day 4 was chosen, anticipating that surgical stress was over [2], and that the duration (day 1–3) of hydrocortisone treatment was too short to significantly hamper the HPA axis.

In all patients, postoperative evaluation of the pituitary function was performed after 6–8 weeks, including SST with measurements of S-cortisol before and 30 min after intravenous injection of 0.25 mg Synacthen. A sufficient response was defined as a 30-min S-cortisol concentration above 450 nmol/L [12] .

### Statistical analysis

Descriptive statistics were used for population and group characteristics. Independent *t* test, chi-square, Kruskal-Wallis test, Spearman correlation, cross-tabulation, and ANOVA were used for group comparisons. All data were analyzed and graphically produced with SPSS statistical software version 24.0 (SPSS Inc., Chicago, IL). Statistical significance was set at *P* < 0.05.

## Results

**Fig. 1:**
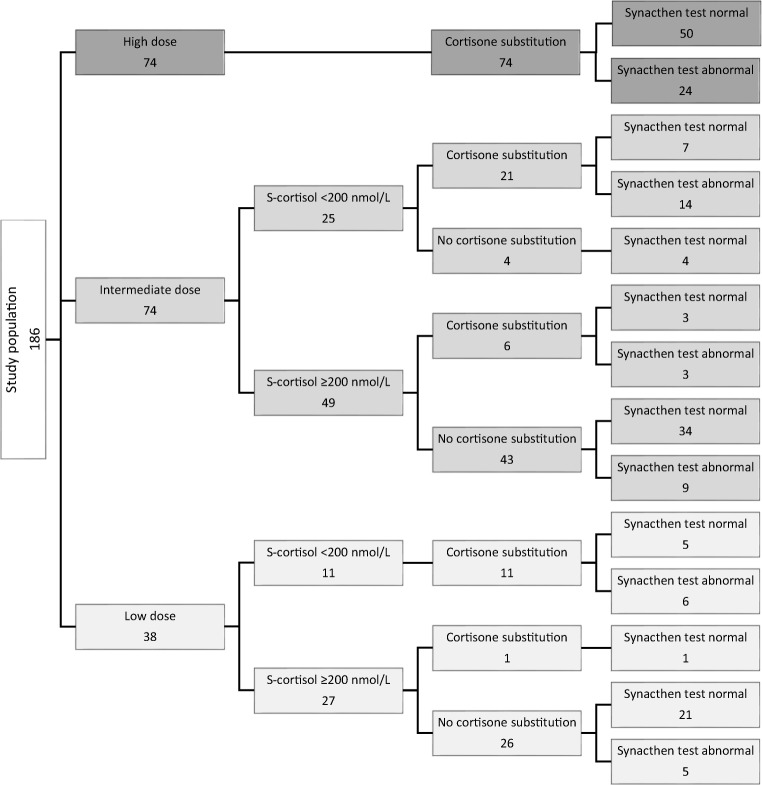
Flowchart of results of SST at follow-up

A total of 186 patients, HD group *n* = 74, ID group *n* = 74, and LD group *n* = 38, were included in the study (Fig. 1). General characteristics are presented in Table 2. There were no significant differences between the groups regarding age or gender. Regarding adenoma type, the groups were also comparable without significant disparity. Slightly fewer microadenomas were included in the LD group.

**Table 2:** General characteristics of the study population

	HD	ID	LD	*P* value
*N*	74	74	38	
Age (years, mean ± SD)	54.9 ± 16	51.6 ± 16	54.5 ± 14	0.397
Females *n* (%)	37 (50)	39 (53)	17 (45)	0.730
Adenoma type				
Inactive (*n*)	44	46	22	
GH (*n*)	8	20	12	
PRL (*n*)	3	3	0	
TSH (*n*)	0	0	1	
Cysts (*n*)	8	5	3	
Micro/macroadenoma	12/62	9/65	2/36	0.252
S-cortisol day 4 (nmol/L, mean ± SD)	–	275 ± 164	349 ± 217	0.047
Cortisone substitution from day 4 to follow-up (*n*)	74	27	12	
Inadequate Synacthen response at follow-up *n* (%)	24/74 (32)	26/74 (35)	11/38 (29)	0.058

### Day 4 S-cortisol

**Fig. 2:**
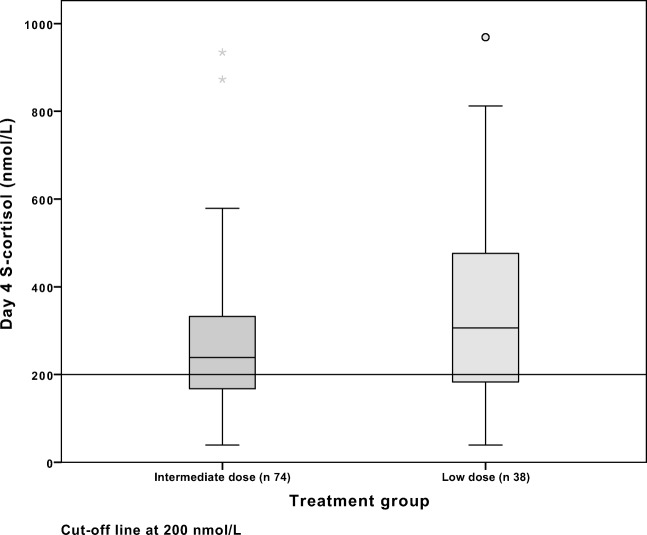
S-cortisol at postoperative day 4, ID and LD groups

The results of S-cortisol at postoperative day 4 are presented in Table 2, Figs. 2 and 4. There was no statistically significant difference between the ID and LD groups regarding the number of patients with S-cortisol at postoperative day 4 with S-cortisol levels below 200 nmol/L. In the ID group, 25/74 (34%) had a S-cortisol below 200 nmol/L day 4 as compared with 11/38 (29%) in the LD group.

**Fig. 3:**
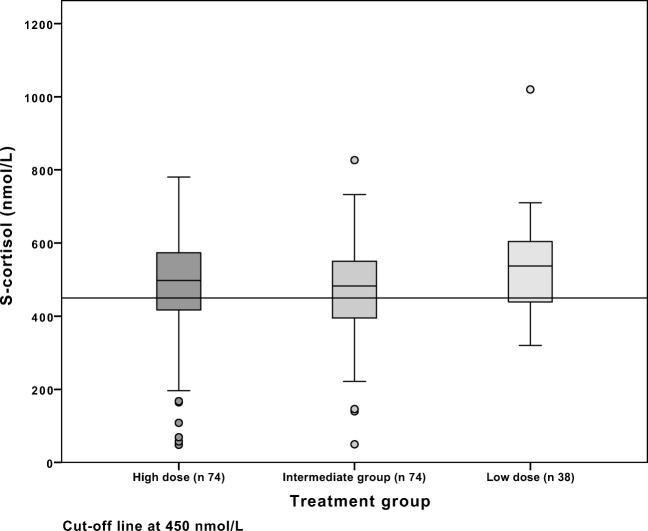
Synacthen response at follow-up by group

**Fig. 4:**
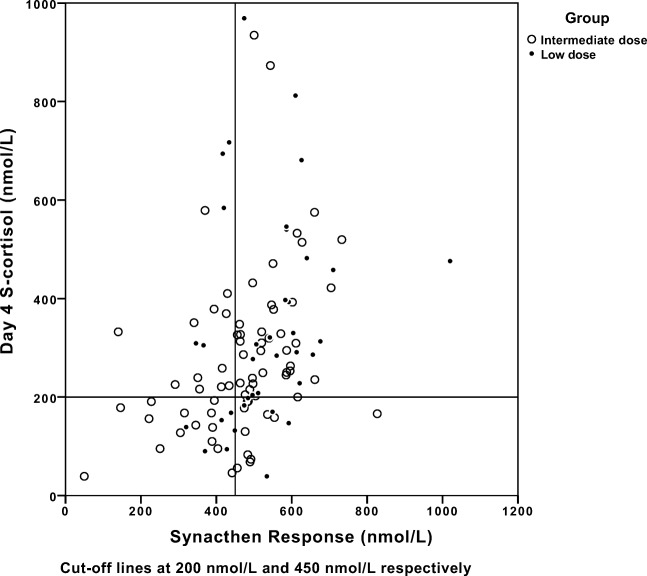
Scatterplot of day 4 serum cortisol and Synacthen response at follow-up

Of the patients with S-cortisol below 200 nmol/L day 4, 14/25 (56%) in the ID group failed SST, as compared with 6/11 (55%) in the LD group. The distribution of day 4 S-cortisol and SST response are presented in Figs. 2 and 3 respectively and the diversity of measurements in Fig. 4.

Similar rates of failure at SST, in patients with S-cortisol above 200 nmol/L, were observed in the ID group 12/49 (24%) and in the LD group 5/27 (19%).

### Short Synacthen test

The results of SST at follow-up are presented by group in Figs. 1 and 4. There was no difference between the groups regarding failure rate of SST. The rate of failed SST (all groups) was 51/186 (27%). Specifically, the failure rate was 24/74 (32%) in the HD group and 26/74 (35%) and 11/38 (29%) in the ID and LD groups respectively. Neither gender nor age did significantly increase the risk of impaired response to SST in any of the groups at follow-up.

Patients who were treated with postoperative hydrocortisone (all groups) until follow-up failed SST in 47/113 (42%). In the HD group 24/74 (32%) failed SST, while 17/27 (63%) and 6/12 (50%) failed SST in the ID and LD groups respectively.

Patients not treated with postoperative hydrocortisone until follow-up (ID and LD groups only), failed SST in 14/73 (19%). In the ID group 9/47 (19%) failed SST, while 5/26 (19%) failed SST in the LD group.

### Relation between short- and long-term evaluation of pituitary function

There was a significant but weak correlation, *r*_s_ 0.330 (*P* < 0.01) between S-cortisol day 4 and SST at 4–6 weeks. The positive predictive value of S-cortisol cutoff level at 200 at day 4 in relation to SST at 4–6 weeks follow-up was 77.6% while the negative predictive value was 55.6%.

## Discussion

In this retrospective study, we found no differences in the number of patients with insufficient response to SST between three groups with markedly different doses of perioperative hydrocortisone treatment after transsphenoidal pituitary surgery. A trend towards a lower rate of patients with S-cortisol less than 200 nmol/L at postoperative day 4 was seen in the group, who received the lowest dose (LD group). The rate of patients with S-cortisol postoperative day 4 above 200 nmol/L was similar in the two groups (ID and LD groups) where this variable was measured. Our study shows that S-cortisol at day 4 above 200 nmol/L predicts a normal SST within 77.6% of the patients in comparison with previous material [17]. Insufficient response to SST at follow-up seemed not to be dependent of perioperative hydrocortisone dose, whereas the rate of patients with S-cortisol below 200 nmol/L did. These results may point towards an inhibitory effect on S-cortisol day 4 related to the dose of perioperative hydrocortisone, showing higher response when the lowest dose (LD) was given.

In our patients, the preoperative function of the HPA-axis was assessed per the patient’s clinical status and the level of morning S-cortisol. The time from diagnostics to elective pituitary surgery is short in our hospital (in general a few weeks) and as all patients undergoing pituitary surgery received hydrocortisone treatment, although using different schedules, no further preoperative biochemical evaluation of the HPA-axis was performed. The size of the tumor and the presence of other pituitary deficiencies were other risk factors to perioperative hypocortisolism, which were considered. A single morning cortisol has been shown to be diagnostic of adrenal insufficiency if the level is below 100 nmol/L [12]. Our results indicate that S-cortisol < 200 nmol/L seems to be an acceptable level to identify patients who need continuous hydrocortisone replacement in accordance with previous publications [9, 14, 16–18]. However, the diversity of S-cortisol at day 4 in our study indicates that, to predict long-term HPA-axis dysfunction at this point, the cutoff level must be significantly increased (Fig. 4). Doing so though, would imply less specificity to the test. We therefore argue that an arbitrary limit of morning cortisol must always be related to the patient’s clinical status. Consequently, some of our patients with suspected symptoms of hypocortisolism continued with hydrocortisone after day 4 despite having levels > 200 nmol/L. The reasons for this were in most cases, suspected or verified postoperative infections, and/or values close to cutoff level.

It is a challenge to balance the risk of hypocortisolism and the risk of overtreatment with hydrocortisone in patients undergoing pituitary surgery. During the period where the HD schedule was used, practically no patients were at risk of hypocortisolism. On the other hand, some patients were treated unnecessarily with hydrocortisone for weeks, and thereby at risk for unwanted side effects of hydrocortisone therapy. In contrast, the use of lower hydrocortisone doses and discontinuing this treatment after a few days might put some patients at risk of hypocortisolism. Low-dose schedules therefore imply the need for a close and thorough monitoring of the patients until the SST is performed. Discontinuation of hydrocortisone is also beneficial considering a simpler postoperative period without the need of hydrocortisone and decisions on changing the dose of it. Use of lower hydrocortisone doses and evaluation on day 4 has, in our experience, simplified the management of patients in the postoperative period after pituitary surgery.

It is a limitation that data was retrospectively collected from the medical records and not from a prospective, systematic controlled study.

In summary, we could not correlate any differences in long-term HPA-axis function in three different hydrocortisone treatment modalities. We found a weak but significant correlation between low day 4 S-cortisol and failed SST at follow-up, in line with the diversity of previous publications [17, 22]. We observed no clinically significant impairment using lower hydrocortisone doses. Our results add to previous knowledge and recommendations, and conclude that an individualized regimen for hydrocortisone treatment is recommended in these patients.
